# Improvements in the Engineering Properties of Cementitious Composites Using Nano-Sized Cement and Nano-Sized Additives

**DOI:** 10.3390/ma15228066

**Published:** 2022-11-15

**Authors:** Ibadur Rahman, Priyanka Singh, Nirendra Dev, Mohammed Arif, Faiz Noor Khan Yusufi, Ameer Azam, M. Masroor Alam, Sandeep Singh, Jasgurpreet Singh Chohan, Raman Kumar, Lovneesh Sharma, Elsayed Tag-Eldin, Shubham Sharma, Muhammad Rizal Muhammad Asyraf

**Affiliations:** 1 Department of Civil Engineering, Jamia Millia Islamia, Jamia Nagar, New Delhi 110025, India; 2 Department of Civil Engineering, Amity School of Engineering & Technology, Amity University Uttar Pradesh, Noida 201313, India; 3 Department of Civil Engineering, Delhi Technological University, Shahbad, Daulatpur, Bawana Road, New Delhi 110042, India; 4 Department of Civil Engineering, Aligarh Muslim University, Aligarh 202002, India; 5 Department of Statistics & Operations Research, Aligarh Muslim University, Aligarh 202002, India; 6 Department of Applied Physics, Aligarh Muslim University, Aligarh 202002, India; 7 Department of Civil Engineering, University Center for Research and Development, Chandigarh University, Mohali 140413, India; 8 Mechanical Engineering Department, University Center for Research & Development, Chandigarh University, Mohali 140413, India; 9 Department of Civil Engineering, Universal Institute of Engineering & Technology, Mohali 140413, India; 10 Faculty of Engineering and Technology, Future University in Egypt, New Cairo 11835, Egypt; 11 School of Mechanical and Automotive Engineering, Qingdao University of Technology, Qingdao 266520, China; 12 Engineering Design Research Group (EDRG), Faculty of Mechanical Engineering, Universiti Teknologi Malaysia, Johor Bahru 81310, Malaysia; 13 Centre for Advanced Composite Materials (CACM), Universiti Teknologi Malaysia, Johor Bahru 81310, Malaysia

**Keywords:** cementitious composites, dry- and wet-grinding, nano-sized cement, nano-sized additives, morphological studies, stepwise multiple regression, repeated measures ANOVA

## Abstract

The findings of an extensive experimental research study on the usage of nano-sized cement powder and other additives combined to form cement–fine-aggregate matrices are discussed in this work. In the laboratory, dry and wet methods were used to create nano-sized cements. The influence of these nano-sized cements, nano-silica fumes, and nano-fly ash in different proportions was studied to the evaluate the engineering properties of the cement–fine-aggregate matrices concerning normal-sized, commercially available cement. The composites produced with modified cement–fine-aggregate matrices were subjected to microscopic-scale analyses using a petrographic microscope, a Scanning Electron Microscope (SEM), and a Transmission Electron Microscope (TEM). These studies unravelled the placement and behaviour of additives in controlling the engineering properties of the mix. The test results indicated that nano-cement and nano-sized particles improved the engineering properties of the hardened cement matrix. The wet-ground nano-cement showed the best result, 40 MPa 28th-day compressive strength, without mixing any additive compared with ordinary and dry-ground cements. The mix containing 50:50 normal and wet-ground cement exhibited 37.20 MPa 28th-day compressive strength. All other mixes with nano-sized dry cement, silica fume, and fly ash with different permutations and combinations gave better results than the normal-cement–fine-aggregate mix. The petrographic studies and the Scanning Electron Microscope (SEM) and Transmission Electron Microscope (TEM) analyses further validated the above findings. Statistical analyses and techniques such as correlation and stepwise multiple regression analysis were conducted to compose a predictive equation to calculate the 28th-day compressive strength. In addition to these methods, a repeated measures Analysis of Variance (ANOVA) was also implemented to analyse the statistically significant differences among three differently timed strength readings.

## 1. Introduction

In the past decade, construction technology has undergone some profound changes. On the one hand, the emphasis has been on the construction techniques, and on the other, on using a variety of construction materials, specifically alternate materials. Many research studies from the viewpoint of both the short- and long-term mechanical characteristics of cementitious composites have been undertaken. This has resulted in the establishment of different additives to improve the physicochemical and mechanical properties of conventional concrete. The neo-improved concrete now shows a much better crack arrest mechanism, better creep–shrinkage performance, high durability, etc. [1,2,3,4]. There are few critical studies on the application of nanotechnology in concrete production. Nano-sized particles, having greater surface area than volume, have a high potential for tremendous chemical activity [5,6,7]. Nano-engineering and nano-modification of cement represent an important arena, though in the embryonic stage as far as mass production and utilization are concerned [7,8,9,10]. A limited number of investigations are available, dealing with the modification of cement in nano-sized particles and their noteworthy effect as nano-binders for concrete [10].

Nanotechnology is another promising field of study (e.g., see Feynman [6]) that is bound to revolutionize material and construction technology when applied to the construction industry [9,10,11,12]. Furthermore, with the increasing demand for optimal material resource realization and sustainable development approaches, research on nanotechnology and its application for improving the performance of cementitious composites are very much warranted [1]. A number of recent studies have demonstrated that the use of cement hydration products on the nano-scale enhances the strength of cementitious composites, and nano-fly ash has been combined with cement matrix in varied quantities in order to collate and compare conventional cement matrix’s engineering features with those of nano-additives [13,14,15]. A significant improvement in the properties of the cementitious matrix was discerned in this study, showing agreement with other studies [16,17,18].

Combining the nano-scale of different materials offers avenues for developing new additives for cement, such as superplasticizers, nano-particles, or nano-reinforcements [16,17,18]. Much of the work to date with nano-particles has been performed with nano-silica (nano-SiO_2_ and nano-titanium oxide (nano-TiO_2_). There are a few studies on incorporating nano-iron (nano-Fe_2_O_3_), nano-alumina (nano-Al_2_O_3_), and nano-clay particles [12,19,20,21,22].

Nano-SiO_2_ is more efficient in enhancing strength and other engineering properties than silica fume. The addition of 10% nano-SiO_2_ with dispersing agents increases the compressive strength of cement mortars by 26% in 28 days compared with only a 10% increase with the addition of 15% silica fume [11,23,24,25]. In this research, nano-cement and nano-silica fumes were developed in the laboratory, and their combined effects were focused on to determine the strength parameters. To the best of our knowledge, this type of work has not been performed previously [24,25,26].

Nano-Al_2_O_3_ shows a significant increase in the modulus of elasticity, i.e., up to 143% at a dosage of 5%. However, it has a limited effect on the compressive strength, and no other significant changes have been reported [21,26,27].

The ability to insert a variety of organic molecules, preferably nano-sized, into the basic C-S-H structure can provide the potential for creative manipulation. This is in addition to the layered structure and the tendency to have structural defects in the silicate chains, such as the missing bridging tetrahedron of C-S-H [28,29,30]. Three schemes for hybridizing, or incorporating, “guest molecules” into C–S–H were proposed by Minet et al. [18] who demonstrated that it could accommodate small-sized organic groups directly linked to the silicate chains in the interlayer space of C–S–H [11,26,31,32].

A recent study by Reza found that “nano-MgO” influences the “microstructure” and “strength” of “cement composites” [31]. By comparing the “treated composites” with the “plain composite”, the findings indicate that, in 7 and 28 days, 1% of “nano-MgO binder” by weight increases the “compressive strength” by 103 and 80%, respectively, while 95 and 70% of the “flexural strength” are the increases for the plain composites. The “microstructure” of the “cementitious composites” with “nano-MgO” is more “compact” and “homogeneous” than that of “plain composites” due to the “expansive properties” of “nano-MgO” [31].

From the existing studies, it is possible to develop novel “multifunctional”, efficient, performance-enhancing “cementitious composites” that can be suited for a wide range of applications by manipulating the “material’s structure” on the “nanoscale”, such as with “nanoparticles” and “nanoscale fibers” [23]. “Conventional concrete” has been shown to be improved in its “pore structure” to form a “C–S–H gel” faster and to be stronger, more “flexible”, and more “durable” when reinforced with “nanoparticles”. The reinforcement characteristics of “nano-additives” and “nano-sized cement” have been demonstrated to enhance the “strength”, “fracture characteristics”, and “durability” of “cementitious composites” [23].

Hence, nanotechnology researchers have begun to develop novel materials in recent years due to the need to use them for increasing the characteristics of various materials in general and materials utilized in the construction sector in particular [33,34,35]. Previous studies have investigated normal cement composites with nano-additives [36,37], whereas this research study focused on converting cement into nano-cement, followed by converting other additives into nano-additives; then, their individual and combined effects were studied.

Hence, nano-sized cement as well as nano-additives have nano-sized characteristics that change “cement hydration”, the “compaction degree”, and the “thixotropy behavior”, contributing to the “modification of cement hydration”. It is possible to explore all of these effects to make “concrete” that is “stronger”, “greener”, and “workable”. In addition to raising the “mechanical properties” of “cement-based composite materials”, the “chemical reactivity” of “nanomaterials” can be used to modify the “morphology”, thus optimizing the “C–S–H gel structure” and improving the “nanomechanical properties”, which have been proved to have high “durability enhancement capabilities”.

According to literary sources, “nano-additives” and “nanosized cement” increase the “packing density” both in the “cementitious matrix” as well as in the “interfacial transition zone”. In the “interfacial transition zone” between the “nano-reinforcements” or “nano-additives” and the “cementitious matrix,” the “friction at the interface” is dependent on “packing density” and “stiffness” [38]. Therefore, a “cementitious composite” that is frictionally bound has superior “mechanical characteristics.” Several studies have found that “nano-additives” and “nanosized cement” accelerate “hydration,” thereby contributing to the development of “flexural strength” in the early stages. It is also worth noting that lower “CH content” and higher “C–S–H content,” along with the “seeding effect” of “nano-sized particles,” extensively contribute to strengthening the “interfacial-frictional bond.”

It was found through a literature search that the utilisation of nanotechnology in cement paste, mortar, and concrete is on the rise. This growth can be attributed to the availability of freshly discovered nanomaterials, as well as the rising popularity of nanomaterial-modified cement composites. Nanotechnology in cements focuses on two key areas: the creation of novel nano-scale-engineered products for the concrete industry and the characterization and understanding of materials on the nano- and micro-scales using cutting-edge characterization techniques. Hitherto, limited studies can be found where comparisons are made between the dry- and wet-grinding of the constituents. Moreover, the literature survey reported a lack of investigations into the mechanical influence of different permutations and combinations of nano-sized dry cement, silica fume, and fly ash. Thus, the influence of these nano-sized cements, nano-silica fumes, and nano-fly ash in different proportions was studied to evaluate the engineering properties of cement–fine-aggregate matrices using normal-sized, commercially available cement.

## 2. Materials and Experimental Programme

To assess the effect of nano-modification on cement matrices, nano-sized dry- and wet-ground cements were produced and were used as additives in cement matrices to test their strength. Other construction materials used in different proportions in the mix were cement, fine aggregates, coarse aggregates, silica fumes, fly ash, and acetone.

Cement, fine aggregates, silica fume, and fly ash were studied using a standard petrographic microscope and a Scanning Electron Microscope (SEM). Nano-cement was also studied under a Transmission Electron Microscope (TEM), as well as a SEM. Finally, cement concrete blocks were cast, and their compressive strength was measured as per the recommendation of the relevant Indian Standard Code of Practices. It is necessary to evaluate the compressive strength of cement mortar cubes in order to determine whether or not cement satisfies the requirements of the Indian standard specifications and whether or not it is capable of producing concrete with the necessary compressive strength.

Concrete is utilised in construction because of its ability to withstand compressive stresses. However, in situations where the tensile strength or shear strength is of major concern, the compressive strength is estimated to be the most important property of a cement mortar cube.

### 2.1. Materials Used for Experimentation

Shree Ultra 43 grade ordinary Portland cements were utilized for the research work. In the laboratory, the physical properties of cement were determined, as indicated in Table 1.

Badarpur sand was selected since it was readily accessible. The sand was separated and screened to remove lumps of clay and other foreign and detrimental materials, after which it was washed with water and air-dried. Table 2 shows the grading and fineness modulus of sand.

$$Fineness\;Modulus=\frac{250.35}{100}=2.5035$$

Fly ash was collected from the Panipat (Haryana) thermal power plant. The physical properties of fly ash are given in Table 3.

**Figure 1:**
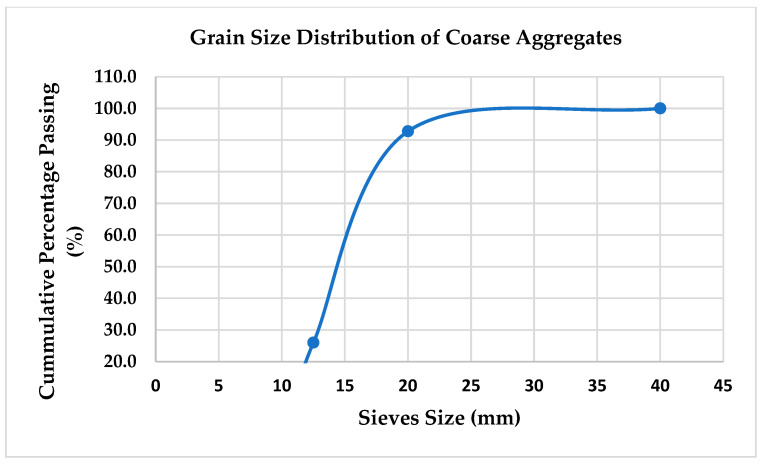
Grain size distribution of coarse aggregates.

Coarse aggregates of locally available quartzite were used in the mix. The properties of the coarse aggregates were determined via sampling and testing in accordance with the requirements of BS 812: Part 103:1985. The specific gravity of the aggregates was 2.76, and the water absorption was 0.40 percent. The particle size distribution of the coarse aggregates used was as presented in Table 4 and illustrated in Figure 1.

For the experiments, we obtained silica fume from Elkem Company. The properties of silica fume are given in Table 5.

### 2.2. Conversion of the Constituent Material to Nano-Size

**Figure 2:**
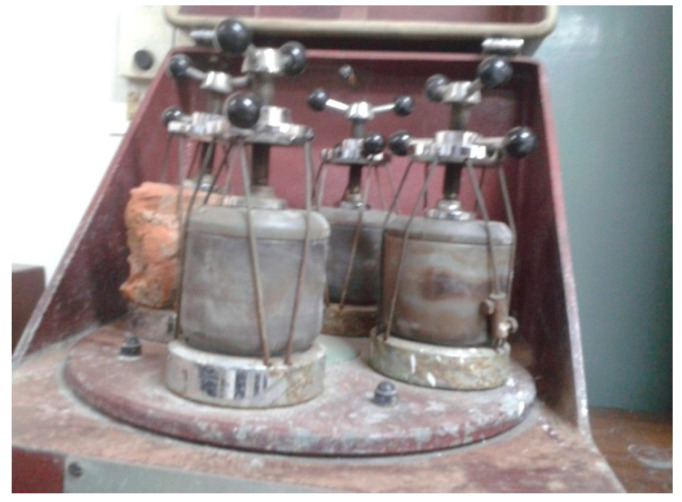
Inside view of ball mill machine.

**Figure 3:**
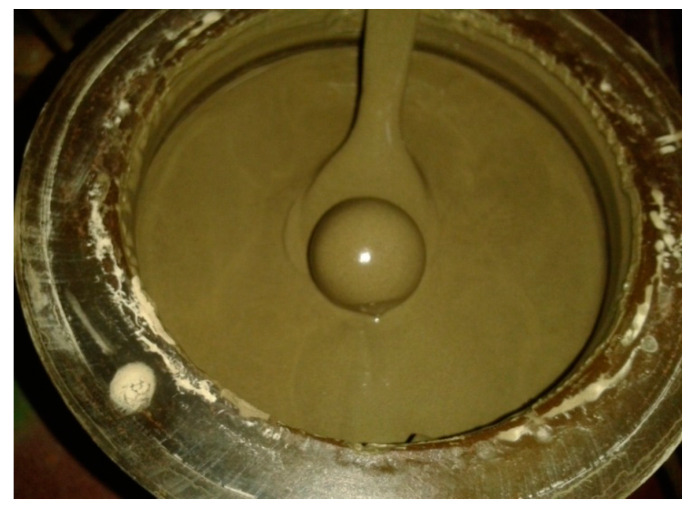
Inside view of wet-grinding of cement.

The conversion of the constituent particles to the nano-scale was carried out with the help of Ball Mill Machine (FRITSCH, Germany) in two ways, i.e., via dry-grinding (Figure 2) and wet-grinding (Figure 3). Dry-grinding was carried out for all the constituents, while wet-grinding was only carried out for cement. Dry-grinding reduces the size via particle-on-particle impacts, while wet-grinding smashes particles against solid grinding media in a liquid, dispersing the raw material in a slurry and then circulating it. In areas with ample water supply, contractors are naturally inclined to use concrete wet-grinding and -polishing, since they were developed long before dry-grinding. Throughout this process, water is primarily used to prevent the diamond tool from overheating and to provide lubrication to reduce friction. It is generally more efficient to grind wetly. This is because thorough mixing takes place when the material is mixed, enabling more balanced feed to be sent directly to the grinding mill. This hazard is eliminated, since dust is not prevalent. This results in a cleaner plant. It is possible to perform a more efficient classification for kiln feed, although thickeners are required due to high dilutions. It is less expensive to resort to less efficient dry-grinding methods where low-cost fuel is available, because the extra heat needed during calcining drives off the water.

Due to the development of better air separators and dust collectors, some of these problems have been minimized to the point where present-day costs are virtually identical.

**Figure 4:**
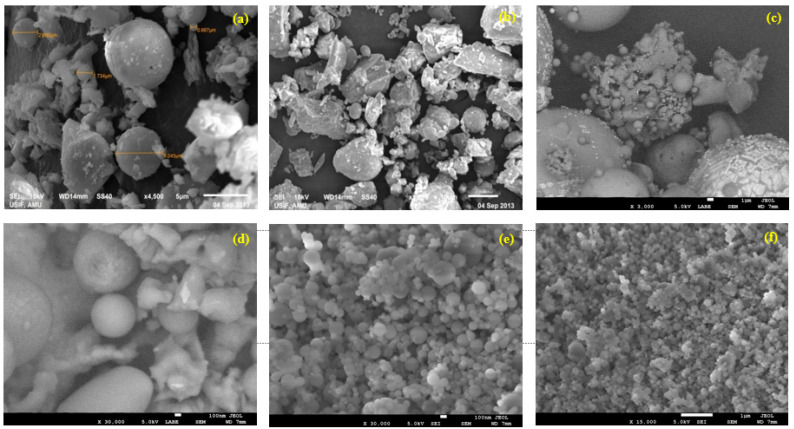
SEM images of (**a**) normal cement, (**b**) converted nano-cement, (**c**) fly ash, (**d**) nano-fly ash, (**e**) silica fume, and (**f**) nano-silica fume.

**Figure 5:**
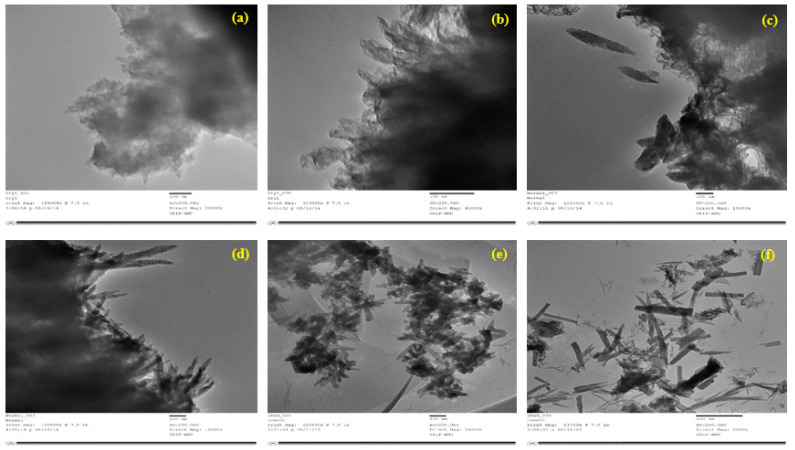
TEM images of (**a**) normal cement, (**b**) normal cement (another view), (**c**) dry-ground nano-cement, (**d**) dry-ground nano-cement (another view), (**e**) wet-ground nano-cement, and (**f**) wet-ground nano-cement (another view).

Acetone was utilized for the wet-grinding of cement. All the converted materials were studied under a Scanning Electron Microscope (SEM) before and after conversion to ascertain the morphological properties of the particles of cement, fly ash, and silica fumes, and the results are presented in Figure 4, respectively. Transmitted electron microscopy was also used for ordinary cement, dry-ground cement, and wet-ground cement, and the results are shown in Figure 5, respectively. Numerous analytical attachments, including multiple EDS, EBSD (co-planar with EDS), WDS, CL, STEM, heating/cooling sub-stages, etc., may be easily mounted onto the SEM (Jeol 700SM) large specimen chamber through ports carefully located on the instrument. Similarly to the SEM, the TEM is an instrument used for electronic spectroscopic imaging. The TEM has the capacity to perform analytical measurements and has a higher spatial resolution than the SEM.

### 2.3. Compressive Strength Test of Cement Matrix

**Figure 6:**
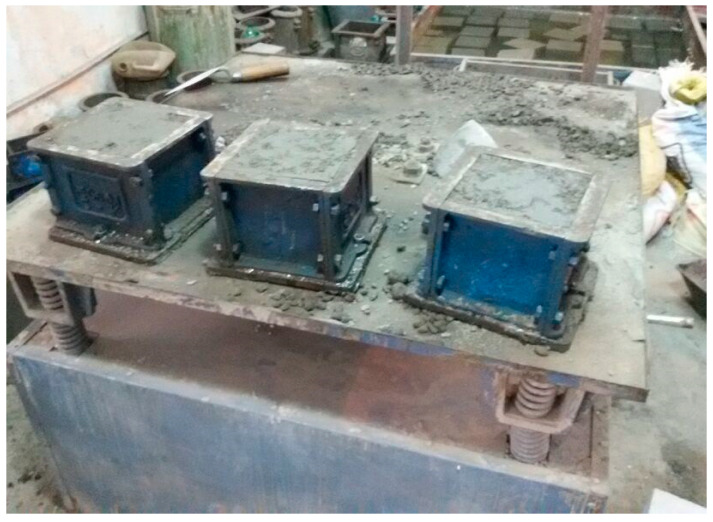
Concrete specimen in the mould on vibrating table.

To study the materials’ performance and mix, 70.06 mm size cubes were cast in the laboratory (Figure 6). In total, 176 cement cube samples were cast, and specific designations were assigned to them. The details of the work plan are given in Table 6. The constituent materials of the cubes were cement, fine aggregates, and potable water. The ratio of cement to fine aggregates was taken as 1:3 by weight. The water–cement ratio was kept as 0.45. The proportions of the material matrices included a fixed sand proportion, i.e., 3, and variations in the cement content, i.e., from 0.45 to 1, in the five materials (i.e., nano-cement, silica fume, nano-silica fume, nano-fly ash, and fly ash), forming binary, tertiary, and quaternary blends of the cementitious mixture. The fractional variations of cement and nano-cement were 0.1, 0.45, 0.5, and 1, respectively, whereas the shares of silica fume, nano-silica fume, fly ash, and nano-fly ash were kept fixed, i.e., 0.1 only. After casting the specimens, the cubes were cured for the specified durations of 7, 14, and 28 days. To determine their crushing strength, the cubes were put through compression testing equipment until they failed.

The compressive strength of the cement-paste cubes is given in Table 7. The cement matrix cubes including the nano-particles demonstrated outstanding strength after 7, 14, and 28 days, as can be observed. As a result, the material system could be trusted for its intended use.

### 2.4. Compressive Strength Test on Concrete

To study the performance of concrete, cubes of the size of 150 mm × 150 mm × 150 mm × 150 mm were cast in the laboratory. A total of six concrete cube samples were cast with the specific designation assigned to these. The detailed work plan is given in Table 8. The constituent materials used for the concrete cubes were cement, fine aggregates, coarse aggregates, and water. M20 grade nominal mix was used. In this case, the water-to-cement ratio was maintained as 0.50. These cubes were demoulded after 24 h and were allowed to cure in the curing tank for a total of seven days. The cubes were subjected to compressive testing in the laboratory under compression testing equipment until they failed in order to determine their crushing strength. According to the results in Table 9, the compressive strength of concrete cubes was good after seven days, indicating that the concrete cubes including the nanomaterials had excellent strength after seven days (Figure 6).

### 2.5. Microscopic Examination of Cement Matrix

**Figure 7:**
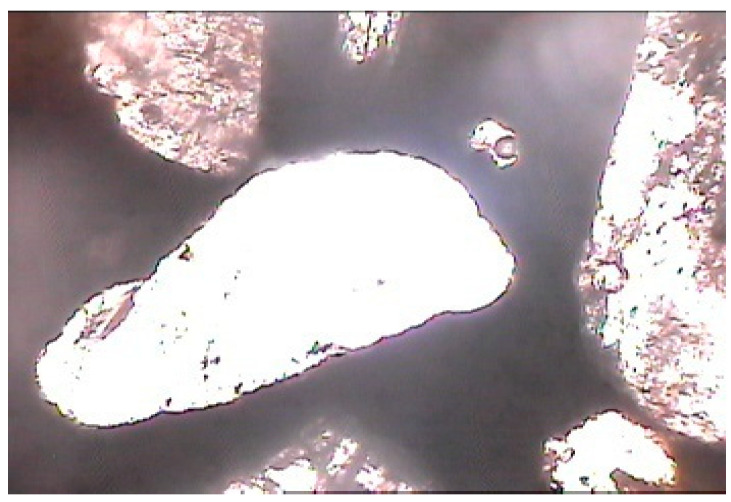
Photomicrograph of normal cement–fine-aggregate mix showing micropores within cement and loose contact between cement paste and aggregate grains (magnification of 60× under non-polarized light).

**Figure 8:**
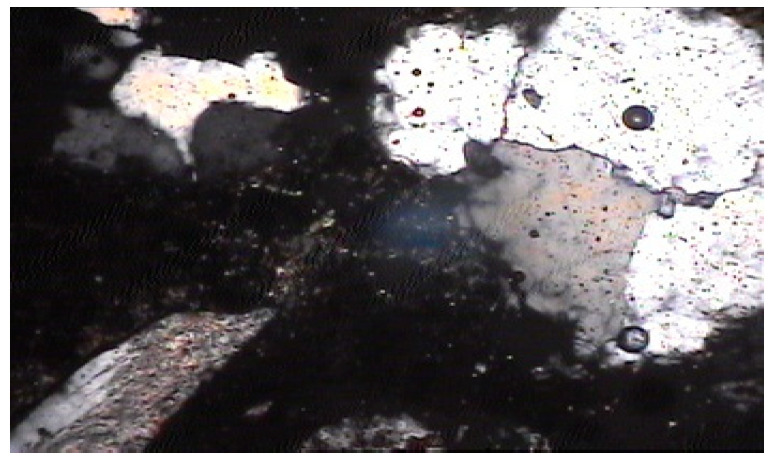
Photomicrograph of normal cement–fine-aggregate mix showing micropores within cement and loose contact between cement paste and aggregate grains (magnification of 60× under polarized light).

**Figure 9:**
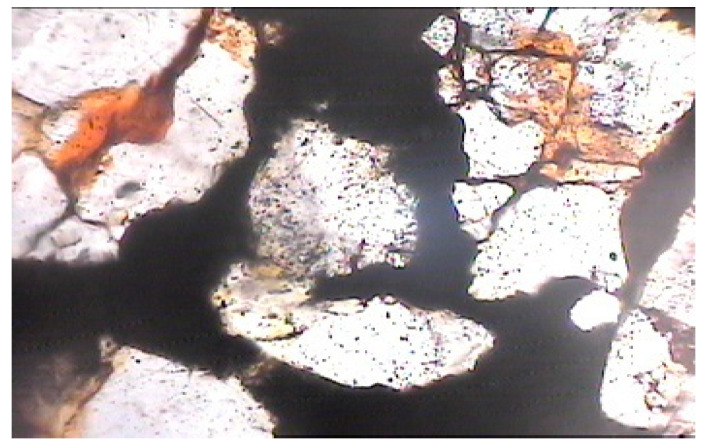
Photomicrograph of mix of nano-sized dry-ground cement and fine aggregates showing dense cement phase and good contact between cement paste and aggregate grains (magnification of 60× under non-polarized light).

**Figure 10:**
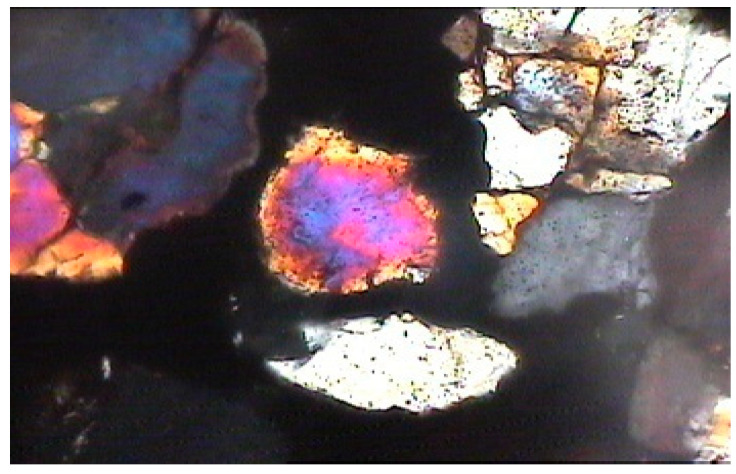
Photomicrograph of mix of nano-sized dry-ground cement and fine aggregates showing dense cement phase and good contact between cement paste and aggregate grains (magnification of 60× under polarized light).

**Figure 11:**
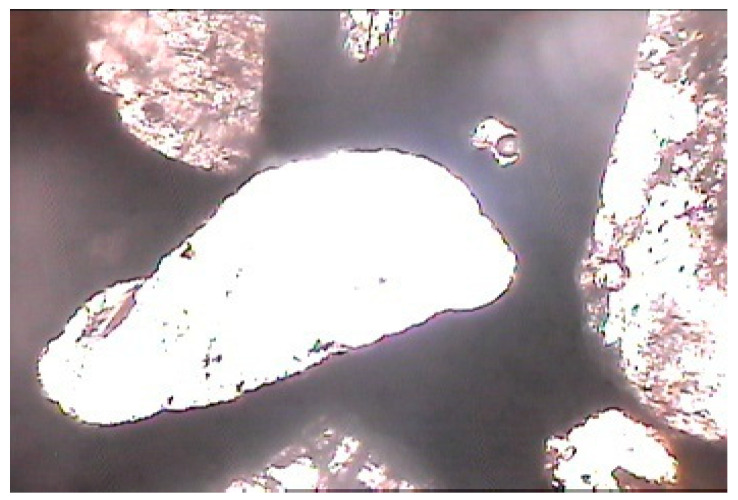
Photomicrograph of mix of nano-sized wet-ground cement and fine aggregates showing dense cement phase and good contact between cement paste and aggregate grains (magnification of 60× under non-polarized light).

**Figure 12:**
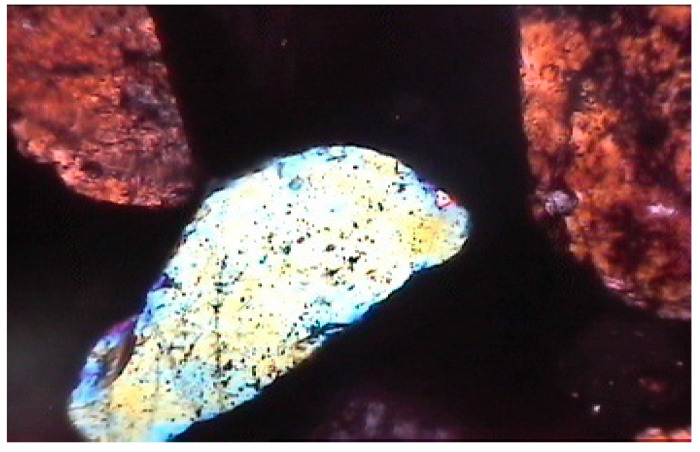
Photomicrograph of mix nano-sized wet-ground cement and fine aggregates showing dense cement phase and good contact between cement paste and aggregate grains (magnification of 60× under polarized light).

The cement cubes, cast and tested using a universal testing machine, were subjected to petrographic studies under a microscope [22,23,24,25]. The pieces of the cube after testing were converted into thin sections for studying under a petrological microscope [26,27,28,29]. The slides of different types of cement cubes were prepared and studied under normal and polarized light. The petrographic studies of cements began with transferring a small amount with a needle to refractive index oil on a clean glass slide for the inspection of individual grains (oil immersion mounts). After that, grains were sprayed on an epoxy-coated frosted glass slide for petrographic thin-section investigation. The representative sample was then encased with epoxy in a castable mould for thin sectioning and petrographic analyses. Then, polished sections were obtained for scanning electron microscopy, and a small bulk sample (10 grammes) was quartered for X-ray diffraction or chemical analyses. It was found that concrete chips were denser when they included nano-sized cement [33,34,35]. The effect of dry- and wet-ground cements on the matrices could not be found out on this scale of magnification. The following were analysed:

- Normal cement cubes (Figure 7 and Figure 8);
- Dry-ground cement cubes (Figure 9 and Figure 10);
- Wet-ground cement cubes (Figure 11 and Figure 12).

### 2.6. Statistical Analysis

Several statistical techniques were applied on the compressive strength values of different material compositions to statistically justify our study findings and create a predictive model using the variables [36,37,38].

A repeated measures ANOVA was implemented to test the difference between the compressive strength readings collected after 7, 14, and 28 days. A *p*-value below 0.05 defined significance among the groups [39,40,41]. Pearson correlation and stepwise multiple regression analyses were also executed to develop a predictive equation, with strength on the 28th day as the dependent variable and strengths on the 7th and 14th days as two categorical variables, while the type of grinding and a grouping variable were taken as the independent variables. Grouping was defined as the type of mixing, and the grinding variable was categorized as dry, wet, or standard [42,43,44]. Group 1 denotes the composition of cement and sand. Group 2 is the mixture of cement, sand, and silica fume. Lastly, Group 3 is the composition of cement, sand, and fly ash.

## 3. Results and Discussion

This paper presents a discussion of the test findings in order to derive both qualitative and quantitative inferences. Nanotechnology has begun to develop novel materials in recent years due to the need to use them for increasing the characteristics of various materials in general and materials utilized in the construction sector in particular [45,46,47,48]. The results obtained from compressive tests on cement samples are given in Table 6. Previous studies have investigated normal cement composites with nano-additives, whereas this research study focused on converting cement into nano-cement, followed by converting other additives into nano-additives; then, their individual and combined effects were studied [48,49,50]. Previous literature works did not study the nano-cement effects on the composites [51,52,53].

### 3.1. Compressive Strength of Cement Matrix

The results of 176 cement cube specimens of size 70.7 mm are given in Table 6. The graphs show variations mainly where the nano-cement and nano-construction materials with aggregates were used. The variations are discussed in detail below.

#### 3.1.1. Test Results of Cubes with Cement and Nano-Cement

**Figure 13:**
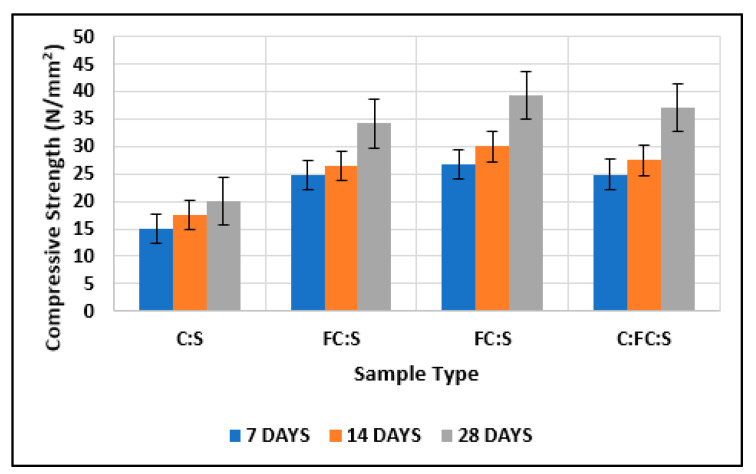
Comparison of test results of cubes with cement and fine cement.

As shown in Figure 13, the compressive strength of nano-cement (converted cement) after 7, 14, and 28 days was greater than the compressive strength of normal cement. Nano-cement (wet-ground) showed greater strength than nano-cement (dry-ground) and normal cement [54,55,56]. The percentage increases in the strength of wet-ground cement compared with normal cement after 7, 14, and 28 days were 76, 75, and 90%, respectively.

In addition, the percentage increases in the strength of dry-ground cement compared with normal cement after 7, 14, and 28 days were 59, 55, and 65%, respectively. The strength of cubes made of cement and fine aggregate lies in the strength of cubes made of nano-cement matrix and normal cement matrix. The fineness of cement, which causes a greater surface area of cement to come into contact with water at successive phases, is responsible for the increase in strength in each step [57,58]. Furthermore, because there are fewer voids, the strength of the structure increases because cracks require space or gaps to propagate through [58,59].

#### 3.1.2. Comparison of Test Results of Cubes with Cement, Nano-Cement, and Silica Fume with Those of Cubes with Cement, Nano-Cement, and Nano-Silica Fume

**Figure 14:**
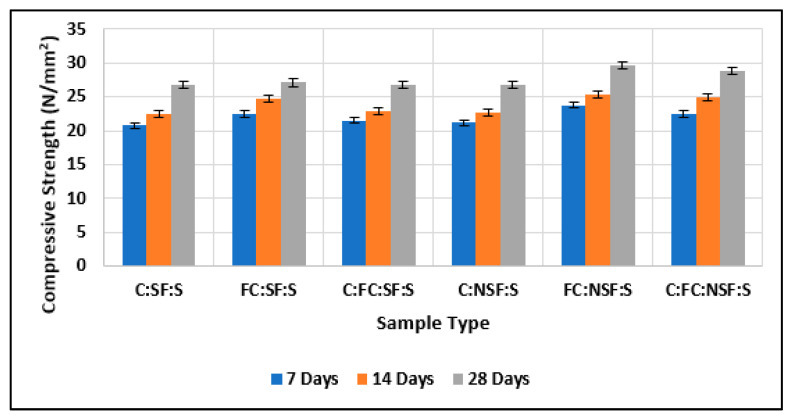
Comparison of test results of cubes with cement, fine cement, and silica fume with test results of cubes with cement, fine cement, and nano-silica fume.

Figure 14 shows the compressive strength comparison of samples with the addition of 10% nano-silica fume and 10% normal silica fume. The graph shows that nano-cement having 10% nano-silica fume showed greater compressive strength than nano-cement having 10% normal silica fume. In addition, nano-cement with 10% nano-silica fume had greater compressive strength than normal cement with 10% normal silica fumes, and the findings depict it to be superior to the results of existing studies [60,61,62].

#### 3.1.3. Comparison of Test Results of Cubes with Cement and Nano-Cement with Test Results of Cubes with Cement, Nano-Cement, and Silica Fume and of Cubes with Cement, Nano-Cement, and Nano-Silica Fume

**Figure 15:**
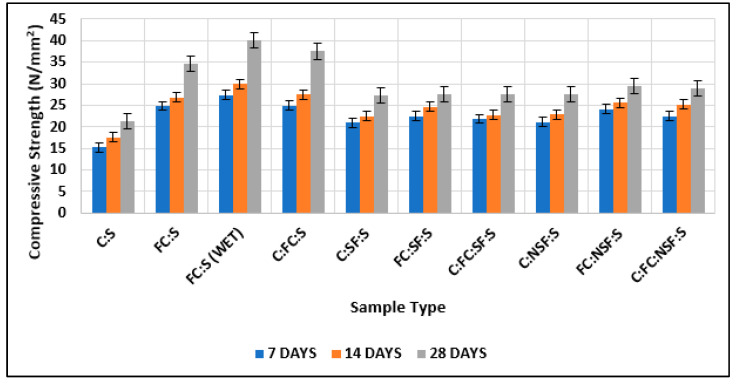
Comparison of test results of cubes with cement and fine cement with test results of cubes with cement, fine cement, and silica fume and with test results of cubes with cement, fine cement, and nano-silica fume.

In Figure 15, it can be seen that the maximum strength was shown by the nano-cement matrix and the cement matrix that had nano-cement and nanomaterials, and the findings are superior to those of existing studies [63,64,65].

#### 3.1.4. Comparison of Test Results of Cubes with Cement, Nano-Cement, and Fly Ash with Those of Cubes with Cement, Nano-Cement, and Nano-Fly Ash

**Figure 16:**
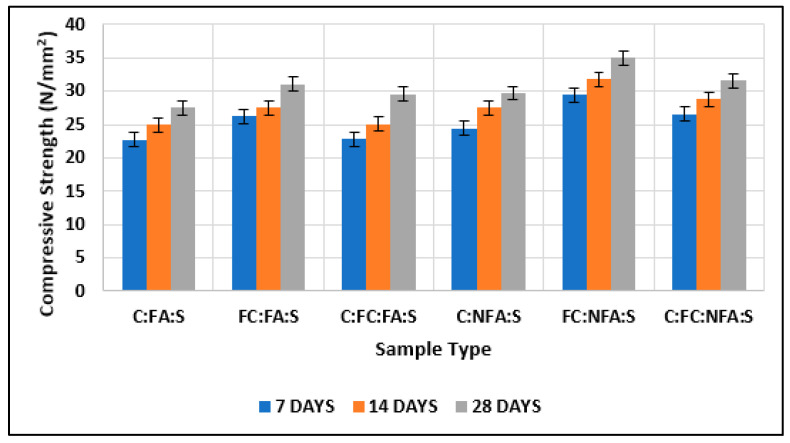
Comparison of test results of cubes with cement, fine cement, and fly ash with test results of cubes with cement, fine cement, and nano-fly ash.

Table 7 and Figure 16 compare the compressive strength obtained by adding 10% nano-fly ash and 10% normal fly ash. This showed that nano-cement containing 10% nano-fly ash had greater compressive strength than nano-cement containing 10% regular fly ash. In addition, nano-cement with 10% nano-fly ash showed greater compressive strength than normal cement with 10% fly ash, and the related findings have been proved in numerous literature studies [66,67,68].

#### 3.1.5. Comparison of Test Results of Cubes with Cement and Nano-Cement with Those of Cubes with Cement, Nano-Cement, and Fly Ash and of Cubes with Cement, Nano-Cement, and Nano-Fly Ash

**Figure 17:**
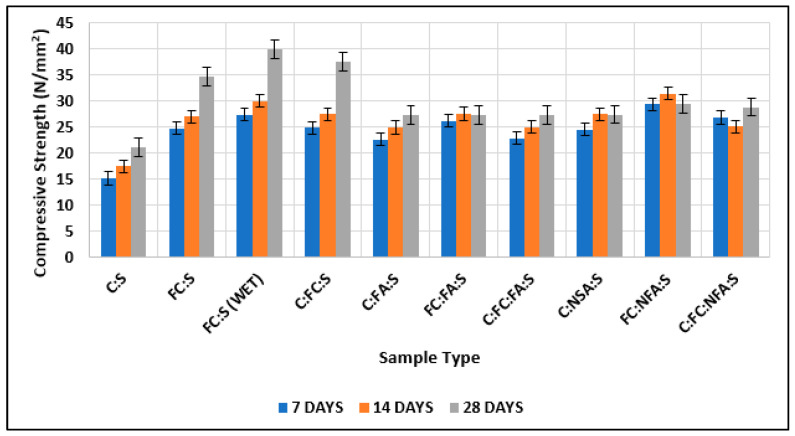
Comparison of test results of cubes with cement and fine cement with test results of cubes with cement, fine cement, and fly ash and with test results of cubes with cement, fine cement, and nano-fly ash.

The maximum strength was shown by the nano-cement matrix and the cement matrix with nano-cement and nanomaterials, as shown in Figure 17. Wet-ground cement attained the maximum value, and the normal cement matrix attained the minimum, which is somewhat related to the results of existing studies [69,70,71,72].

#### 3.1.6. Overall Comparison of Test Results of Cubes with Cement, Nano-Cement, Silica Fume, Nano-Silica Fume, Fly Ash, and Nano-Fly Ash

**Figure 18:**
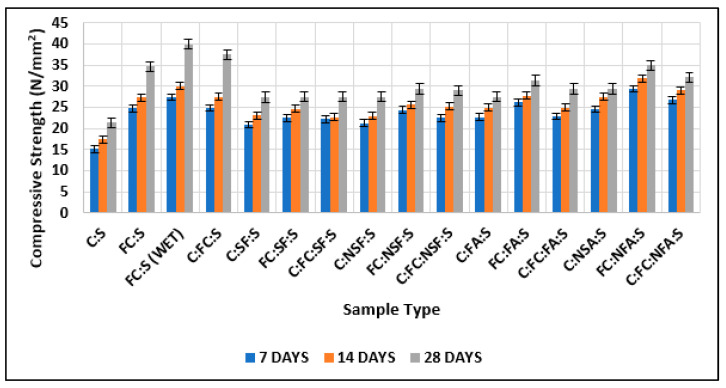
Comparison of test results of cubes with cement (C:S) and fine cement (FC:S) with the following: test results of cubes with cement (C:FC:S), fine cement, and silica fume; test results of cubes with cement, fine cement, and nano-silica fume; test results of cubes with cement, fine cement, and fly ash; test results of cubes with cement, fine cement, and nano-fly ash; and test results of cubes with cement.

Figure 18 shows the summary of all the tables. We can easily conclude that the matrix gained the maximum strength with nano-cement, nano-silica fume, and nano-fly ash. In this research work, the focus was on the comparison of the strength parameters of normal cement composites with those of nano-cement composites. This work can be extended in the future to study the effect of nano-additives, including particle shape, impurity, defects, etc., within cement matrices.

### 3.2. Compressive Strength of Concrete Matrix

**Figure 19:**
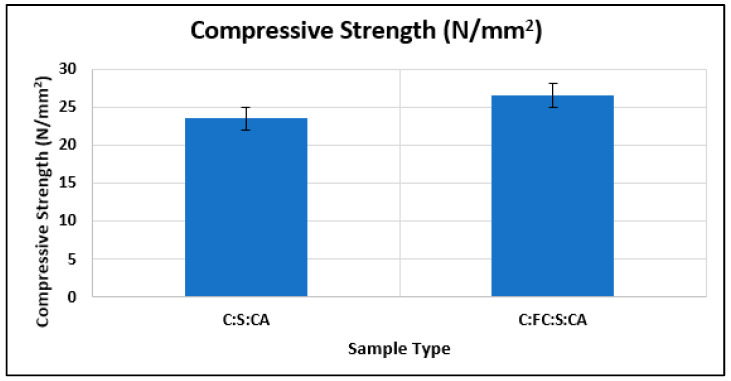
Comparison of test results of concrete cubes with cement and 25% fine cement.

From Figure 19, it is clearly visible that the concrete cubes that contained only 25% wet-ground cement had greater compressive strength than the concrete cubes with normal cement for the M20 mix design.

Since the nano-particles fill the gaps among cement grains and consume a portion of the calcium hydroxide present, the production of extra calcium silicate hydrate (C-S-H) results in an improvement in the interface structure over and beyond what has been previously achieved. The increase in strength can be attributed to the fineness of cement, which allows a higher surface area of cement to come into contact with water and denser micro-fabric to be formed, limiting the number and size of voids, thus improving strength [72,73,74]. The properties of nano-sized wet-ground cement and fine aggregates were comparable to those of nano-sized dry-ground cement. As a result, no distinction could be detected at the petrographic microscope magnification level. Though transmission electron microscopy, we could distinguish the two in terms of the growth of interfingering crystal mesh [75,76,77].

The matrix with nano-cement, nano-silica fume, and nano-fly ash gained the most strength and was shown to have substantial improvement in terms of the compressive strength of cementitious composites after 7–28 days of curing.

### 3.3. Microscopic Examination of Cement Matrix

From the general photomicrographic studies, it seemed that micro-pores were evident within the cement paste in the case of normal cement. The contacts with the aggregate grains were also not very dense (Figure 7 and Figure 8).

In nano-sized dry-ground cement, the mix showed a dense cement phase and good cement–grain contact (Figure 9 and Figure 10). Nano-sized dry-ground cement, in some places, showed that rounded pores had formed due to air tramping in the mix.

The mix made of nano-sized wet-ground cement and aggregates showed characteristics similar to those of nano-sized dry-ground cement [76,77,78]. As such, no differentiation could be made at the magnification level of petrographic microscopy (Figure 11 and Figure 12). Detailed scanning electron microscopy may help to unravel the difference between the two. However, the TEM studies on cement showed the development of nanorods with increasing length and slenderness in dry- and wet-ground nano-cement compared with normal cement, which may have played an essential role in the hydration and solidification process involved in the hardening of cement [77,78]. The densifying properties of nano-composites resulted in the reduction in pores in the cement matrix, which enhanced the overall strength. This was evident in the photomicrographic studies. These findings explain the relationship between the strength and the microscopic structure of the cementitious composites.

However, Y.F. Ling et al., Y.R. Jeng et al., and L. Wei et al. have provided comprehensive reviews on the effect of grain size and orientation on the mechanical strength of crystalline nanomaterials [76,77,78,79].

### 3.4. Statistical Findings

Since the compressive strength readings were measured repeatedly in three different periods, the three readings were dependent on each other; hence, there was a strong correlation among them [78,79]. The correlation coefficient between the 7th- and 14th-day strength was 0.986 (*p*-value < 0.05); that between the 7th- and 28th-day strength was 0.828 (*p*-value < 0.05); that between the 14th- and 28th-day strength was 0.882 (*p*-value < 0.05).

The final model consisted of two independent variables, the 14th-day compressive strength and the grouping variable, for the implementation of stepwise multiple regression analyses. These two factors were the only significant variables that the 28th-day strength depended on (*p*-value <0.05). The 7th-day strength and grinding factors were not significant. The regression equation is given below, and this is justified by a very strong, adjusted R-squared value of 93.8%.

28th day compressive strength = 1.391 ∗ 14th day compressive strength—2.483 ∗ Group—0.158

With the help of the above equation, it is possible to calculate the 28th-day compressive strength, and only the 14th-day strength and the type of composition are required.

### 3.5. Repeated Measures ANOVA

The assumptions of equal variance and equal covariance for applying the repeated measures ANOVA test were satisfied. Box’s test of equality of covariance matrices, Levene’s test of equality of variance, and Mauchly’s test of sphericity were all non-significant (*p*-value > 0.05) [77,78,79]. The repeated measures ANOVA method resulted in a significant difference between the three compressive strength readings (*p*-value <0.05) for the group and the grinding categories. The exact difference was identified using Scheffe’s multiple comparison post hoc test. There was a significant difference among all three types of methods in the case of the grinding factor: standard, wet, and dry (*p*-value < 0.05). However, the difference was only found between Group 1 and Group 2 (*p*-value < 0.05) for the group variable. Within-group differences were also tested, and a significant difference was found among the three groups and in the grinding process.

## 4. Conclusions

The following findings were reached as a result of experimental research: The use of nano-additions improved the characteristics of cement matrix and concrete. Because nano-particles filled the gaps between cement grains and consumed a portion of the calcium hydroxide present, the production of extra calcium silicate hydrate (C-S-H) resulted in an improvement in the interface structure over and beyond what has been achieved previously. The increase in strength can be attributed to the fineness of cement, which allows a higher surface area of the cement to come into contact with water and denser micro-fabric to be formed, limiting the number and size of voids, thus improving strength. The properties of nano-sized wet-ground cement and fine aggregates were comparable to those of nano-sized dry-ground cement. As a result, no distinction could be detected at the petrographic microscope magnification level. Though transmission electron microscopy, the two could be distinguished in terms of the growth of interfingering crystal mesh.

The matrix with nano-cement, nano-silica fume, and nano-fly ash gained the greatest strength and was shown to have substantial improvement in terms of the compressive strength of cementitious composites after 7–28 days of curing.

All of the varied mixes using nano-sized dry cement, silica fume, and fly ash in various permutations and combinations provided strength values ranging from 26.75 to 35.20 MPa, which were greater than that of the typical cement–fine-aggregate mix (20.88 MPa). The 50:50 combination of normal and wet-ground cement produced a compressive strength of 37.20 MPa after 28 days. When comparing cube specimens utilizing wet-ground nano-cement (40 MPa) with cube specimens using conventional cement, an increase of up to 92 percent was found (20.88 MPa).

A strong correlation was found among the three compressive strength readings, and a linear equation was composed to calculate the 28th-day compressive strength. A significant difference was identified among the three strength values for the group and the grinding categories.

## Figures and Tables

**Table 1:** Properties of cements.

S. No.	Type of Cement	Consistency	Initial Setting Time (min)	Final Setting Time (min)
1.	Normal cement	28.50%	61	294
2.	Dry-ground nano-cement	30.00%	58	291
3.	Wet-ground nano-cement	32.00%	54	290

**Table 2:** Sieve analysis results of fine aggregates (sand).

S. No.	IS: Sieve Designation	Weight Retained (g)	Cumulative Weight Retained (g)	% Cumulative Weight Retained
1.	4.75 mm	Nil	0	0
2.	2.36 mm	20	20	1
3.	1.18 mm	235	255	12.75
4.	600 micron	560	815	40.75
5	300 micron	1102	1917	95.85
6.	150 micron	83	2000	100.00

**Table 3:** Physical properties of fly ash.

S. No.	Constituent/Property	Value
1.	Colour	Grey
2.	Percent passing 45-micron sieve	90%
3.	Size of the particle	4.70 × 10^−7^ m
4.	Maximum dry density (MDD)	9.30 kN/m^3^
5.	Optimum moisture content (OMC)	27.5%
6.	Specific gravity	2.02 at 27 °C
7.	Surface area	3060 cm^2^/g

**Table 4:** Gradation and properties of locally available quartzite coarse aggregates.

Sieve No.	Mass Retained (g)	Percentage Retained (%)	Cumulative Percentage Retained (%)	Cumulative Percentage Passing (%)
40.00 mm	0	0	0	100
20.00 mm	330	7.2	7.2	92.8
12.50 mm	3060	66.8	74.0	26.0
10.00 mm	1100	24.0	98.0	2
4.75 mm	92	2.0	100.0	0

**Table 5:** Physical properties of silica fume.

S. No.	Description	Result
1.	Particle size	6.09 × 10^−8^ m
2.	Surface area	14 m^2^/gm
3.	Density	2.2 g/cm^3^

**Table 6:** Work plan for compressive strength test on cement matrix.

S. No.	Type	Composition	Abbreviation	Ratio
1.	I	Cement:sand	C:S	1:3
2.	II	Nano-cement:sand	NC:S	1:3
3.	II	Nano-cement (wet):sand	NC:S	1:3
4.	III	Cement:nano-cement:sand	C:NC:S	0.5:0.5:3
5.	IV	Cement:silica fume:sand	C:SF:S	0.9:0.1:3
6.	V	Nano-cement:silica fume:sand	NC:SF:S	0.9:0.1:3
7.	VI	Cement:nano-cement:silica fume:sand	C:NC:SF:S	0.45:0.45:0.1:3
8.	VII	Cement:nano-silica fume:sand	C:NSF:S	0.9:0.1:3
9.	VIII	Nano-cement:nano-silica fume:sand	NC:NSF:S	0.9:0.1:3
10.	IX	Cement:nano-cement:nano-silica fume:sand	C:NC:NSF:S	0.45:0.45:0.1:3
11.	X	Cement:fly ash:sand	C:FA:S	0.9:0.1:3
12.	XI	Nano-cement:fly ash:sand	NC:FA:S	0.9:0.1:3
13.	XII	Cement:nano-cement:fly ash:sand	C:NC:FA:S	0.45:0.45:0.1:3
14.	XIII	Cement:nano-fly ash:sand	C:NFA:S	0.9:0.1:3
15.	XIV	Nano-cement:nano-fly ash:sand	NC:NFA:S	0.9:0.1:3
16.	XV	Cement:nano-cement:nano-fly ash:sand	C:NC:NFA:S	0.45:0.45:0.1:3

**Table 7:** Comparison of test results of cubes with cement (C) and nano-cement (NC) with test results of cubes with cement, nano-cement, and silica fume (SF); test results of cubes with cement, nano-cement, and nano-silica fume (NSF); test results of cubes with cement, nano-cement, and fly ash (FA); test results of cubes with cement, nano-cement, and nano-fly ash (NFA); and test results of cubes with cement.

Type	Composition	Ratio	W/C Ratio	Grinding Type (Cement)	Compressive Strength, 7 Days (MPa)	Compressive Strength, 14 Days (MPa)	Compressive Strength, 28 Days (MPa)
I	C:S	1:3	0.45	---	15.33	17.33	20.88
II	NC:S	1:3	0.45	Dry	24.33	26.73	34.33
II	NC:S	1:3	0.45	Wet	27.00	30.33	40.00
III	C:NC:S	0.5:0.5:3	0.45	Wet	24.45	27.30	37.20
IV	C:SF:S	0.9:0.1:3	0.45	---	20.50	22.60	26.75
V	NC:SF:S	0.9:0.1:3	0.45	Dry	22.30	24.40	27.00
VI	C:NC:SF:S	0.45:0.45:0.1:3	0.45	Dry	21.70	23. 05	26.80
VII	C:NSF:S	0.9:0.1:3	0.45	---	21.00	23.10	26.90
VIII	NC:NSF:S	0.9:0.1:3	0.45	Dry	23.46	25.50	29.20
IX	C:NC:NSF:S	0.45:0.45:0.1:3	0.45	Dry	22.24	24.63	28.86
X	C:FA:S	0.9:0.1:3	0.45	---	22.70	24.35	27.03
XI	NC:FA:S	0.9:0.1:3	0.45	Dry	26.26	27.45	31.00
XII	C:NC:FA:S	0.45:0.45:0.1:3	0.45	Dry	23.30	24.50	29.00
XIII	C:NFA:S	0.9:0.1:3	0.45	---	24.13	26.75	29.03
XIV	NC:NFA:S	0.9:0.1:3	0.45	Dry	29.20	31.60	35.20
XV	C:NC:NFA:S	0.45:0.45:0.1:3	0.45	Dry	26.33	28.33	31.33

**Table 8:** Work plan of compressive strength test on concrete.

S. No.	Type	Mix Design	Composition	Ratio
1.	XVI	M20	Cement: sand: coarse aggregates	1:1.5:3
2.	XVII	M20	Cement: nano-cement: sand: coarse aggregates	0.75:0.25:1.5:3

**Table 9:** Test results of concrete cubes with cement and 25% nano-cement.

Type	Composition	Ratio	Grinding Type (Cement)	Compressive Strength, 7 Days (MPa)
VI	Cement:sand:coarse aggregates	1:1.5:3	---	23.33
XVII	Cement:nano-cement:sand:coarse aggregates	0.75:0.25: 1.5:3	Wet	26.57

## Data Availability

No data were used to support this study.
